# Characterisation of microRNAs from apple (*Malus domestica *'Royal Gala') vascular tissue and phloem sap

**DOI:** 10.1186/1471-2229-10-159

**Published:** 2010-08-04

**Authors:** Erika Varkonyi-Gasic, Nick Gould, Manoharie Sandanayaka, Paul Sutherland, Robin M MacDiarmid

**Affiliations:** 1 The New Zealand Institute for Plant & Food Research Limited (Plant & Food Research) Mt Albert, Private Bag 92169, Auckland Mail Centre, Auckland 1142, New Zealand; 2 Plant & Food Research Ruakura, Private Bag 3123, Waikato Mail Centre, Hamilton 3240, New Zealand

## Abstract

**Background:**

Plant microRNAs (miRNAs) are a class of small, non-coding RNAs that play an important role in development and environmental responses. Hundreds of plant miRNAs have been identified to date, mainly from the model species for which there are available genome sequences. The current challenge is to characterise miRNAs from plant species with agricultural and horticultural importance, to aid our understanding of important regulatory mechanisms in crop species and enable improvement of crops and rootstocks.

**Results:**

Based on the knowledge that many miRNAs occur in large gene families and are highly conserved among distantly related species, we analysed expression of twenty-one miRNA sequences in different tissues of apple (*Malus *x *domestica *'Royal Gala'). We identified eighteen sequences that are expressed in at least one of the tissues tested. Some, but not all, miRNAs expressed in apple tissues including the phloem tissue were also detected in the phloem sap sample derived from the stylets of woolly apple aphids. Most of the miRNAs detected in apple phloem sap were also abundant in the phloem sap of herbaceous species. Potential targets for apple miRNAs were identified that encode putative proteins shown to be targets of corresponding miRNAs in a number of plant species. Expression patterns of potential targets were analysed and correlated with expression of corresponding miRNAs.

**Conclusions:**

This study validated tissue-specific expression of apple miRNAs that target genes responsible for plant growth, development, and stress response. A subset of characterised miRNAs was also present in the apple phloem translocation stream. A comparative analysis of phloem miRNAs in herbaceous species and woody perennials will aid our understanding of non-cell autonomous roles of miRNAs in plants.

## Background

MicroRNAs (miRNAs) are an abundant class of endogenous, non-coding, short transcripts that play important regulatory roles in plants and animals [1-3]. They often occur in large gene families conserved between distantly related species [4-6]. Approximately 20 families of plant miRNAs are moderately to highly conserved and nearly as many fall into the lowly conserved category [7]. In addition, there are many lineage- and species-specific miRNA families [8-11].

In plants, miRNAs repress gene expression by pairing with near-perfect complementary sequences primarily in the mRNA coding region to guide cleavage and translational repression [12-18]. The majority of known plant miRNA targets encode developmentally important transcription factors [19] or other regulatory and stress-response proteins [20-25]. Mutants impaired in miRNA biogenesis exhibit severe abnormalities, and plants ectopically expressing particular miRNAs or miRNA-resistant versions of their targets exhibit a wide range of phenotypes [26-30].

Plant miRNAs have been detected in a range of organs and tissues of model plants and crop species and often show differential expression among different tissues [31-33]. In addition, miRNAs were detected in the phloem sap of cucurbits [34], lupin [35] and oilseed rape [36,37]. The role of phloem miRNAs is not yet clear. While some miRNAs have demonstrated cell-autonomous expression and effects [38-40], miRNAs in the phloem may have a role in establishing gradients of gene expression necessary for developmental patterning and stress responses [32,41-43]. At least one phloem miRNA has a demonstrated long-distance signalling role in the regulation of the plant nutrient status [44,45]. Identification and characterization of miRNAs in both tissues and the phloem sap thus becomes an important step in developing a complete understanding of the regulation of transcription factors and other key regulatory and stress-response genes.

One of the difficulties of studying the long-distance signalling in plants is access to a true representation of phloem cell contents. Various methods have been used in the past to collect exudate from woody and herbaceous plants. The most common method has been to collect exudate from incisions in the bark of woody plants [46] or to cut stems or petioles of herbaceous plants that lack the typical rapid response to vascular damage [47]. These exudation methods allow the collection of samples enriched for the sieve element (SE) contents; however, there is a high likelihood of contamination with the contents of other cells that are damaged by cutting. In addition to contamination from other cells, incision in the vasculature leads to a rapid fall of turgor pressure, a decrease in local water potential, an influx of water, and dilution of the exudate [48]. The decreased water potential may also cause solute influx from associated cells, such as companion cells and neighbouring phloem parenchyma cells. Finally, rapid changes in pressure may result in release of the SE structural components that are otherwise not translocated in the intact vasculature. The standard practice to minimise contamination is to discard the first drop of exudate from a vascular incision, which will reduce but not completely eliminate the contamination from damaged cells [49]. In contrast to exudation methods that are prone to contamination, phloem-feeding insects, such as aphids, enable sampling of SE sap that is often referred to as the gold standard for phloem, being purest and least subject to sampling artefacts. Aphid stylectomy has been used to detect and characterise proteins and to detect specific mRNAs in the SEs of various plant species [50-54]. Because of the size of the aphid stylet, there is no major change in the pressure and water potential, which in turn minimises water and solute fluxes that are inevitable with incision-based sampling.

The aim of this study was to characterise miRNAs from apple (*Malus *x *domestica*) and to determine which miRNAs potentially translocate over long distances in apple sieve tubes. To date, only a handful of apple miRNAs have been identified and their expression in different apple tissues validated [55]. In this study, we validate expression in apple tissues for eighteen out of twenty-one tested miRNA sequences, some of them shown to be conserved between various plant species [56]. In addition, we test for the presence of miRNAs in the apple SE sap obtained by aphid stylectomy and confirm the presence of a subset of miRNAs in the apple phloem translocation stream. Further, we analyse expression of potential targets for apple miRNAs.

## Results

### Conserved miRNAs are differentially expressed in apple tissues

**Figure 1:**
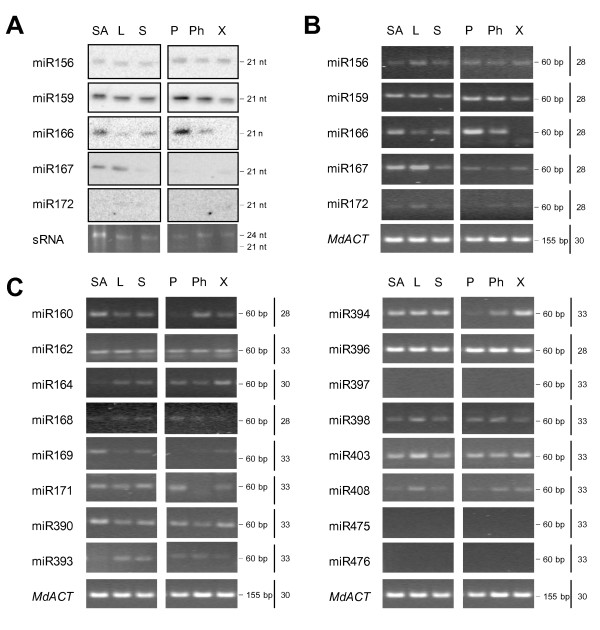
**Differential expression of miRNAs in apple tissues**. A, Gel blot analyses of miR156, miR159, miR166, miR167 and miR172 expression. Low molecular weight RNA purified from 20 μg of total RNA was separated by electrophoresis, transferred and hybridised with an appropriate antisense probe. An ethidium bromide-stained prominent band of small RNA was used as the loading control. B, Stem-loop RT-PCR analyses of miR156, miR159, miR166, miR167 and miR172 expression. 10 ng of total RNA was used in reverse transcription and subsequent PCR amplification. Number of PCR cycles is indicated on the right. Note that 28 cycles of PCR corresponds approximately to gel-blot analysis results. *MdACT *mRNA was amplified using standard RT-PCR. C, Stem-loop RT-PCR analyses using 10 ng total RNA of miR160, miR162, miR164, miR168, miR169, miR171, miR390, miR393, miR394, miR396, miR397, miR398, miR403, miR408, miR475, and miR476 expression. Number of PCR cycles is indicated on the right. *MdACT *mRNA was amplified using standard RT-PCR. SA, shoot apex; L, leaf; S, stem; P, periderm; Ph, phloem; X, xylem (the last three layers were collected by peeling from the stem); sRNA, small RNA loading control; *MdACT*, apple *ACTIN *amplification product.

To establish that the majority of conserved miRNAs are expressed in apple, eighteen miRNAs previously detected in a number of species including *Arabidopsis*, rice (*Oryza sativa*) and poplar (*Populus trichocarpa*), one detected in *Arabidopsis *and poplar but not in rice, and two that were reported to be specific for poplar, were chosen for expression analysis in apple tissues [8,20,57-60] (Table 1). Some miRNAs chosen for this study occur in large gene families with identical and nearly identical sequences. Nearly identical miRNA sequences are given the appropriate miRNA number and a letter suffix that distinguishes between sequences with slight differences [61]. For practical reasons, miRNA sequences with letter suffix 'a' were chosen. These are often the most conserved and most abundant sequences within the miRNA family that were identified in early and relatively small-scale sequencing projects. RNA gel-blot analysis was used to examine the expression of miR156, miR159, miR166, miR167 and miR172 in shoot apex, leaf and stem tissues. Positive signals of expression were detected for most of these miRNAs in either some or all of the tissues (Figure 1A). The relative levels of expression were higher for miR159, miR166 and miR167 than for miR156 and especially miR172, which was barely detectable. miR156 and miR159 were expressed in all tissues tested, with miR156 expressed to similar levels in all tissues, while miR159 levels were somewhat lower in xylem than in other tissues tested. High levels of miR166 expression were detected in the shoot apex and periderm and low levels of expression were detected in leaf, stem and phloem; miR166 was not detected in the xylem. miR167 was highly expressed in the shoot apex and leaf, had low levels of expression in the stem, periderm and xylem and was not detected in the phloem tissue. To establish that stem-loop reverse transcription-polymerase chain reaction (RT-PCR) can be confidently used instead of gel-blot analysis, the expression of the same subset of miRNAs was examined using reactions carried out in a semi-quantitative manner (Figure 1B). The results were comparable to those obtained by RNA gel-blot analysis. Increased sensitivity of this method allowed for detection of low levels of miR172 expression across all tissues analysed. This method confirmed high miR166 expression in periderm, but could not detect any miR166 expression in the xylem tissue, even when the number of PCR cycles was increased from 28 to 35 (data not shown). Further, the stem-loop RT-PCR expression analysis was extended to a larger subset of conserved and poplar-specific miRNAs (Figure 1C). The number of amplification cycles performed ranged from 28 to 33, in order to maintain the semi-quantitative nature of PCR but minimise non-specific amplification. All conserved miRNAs with the exception of miR397 were detectable in some or all examined apple tissues with up to 33 cycles of PCR. At this number of cycles, no amplification or a weak band of primer-dimers was obtained in minus-RT control and water control reactions (data not shown). miR475 and miR476 could not be detected in any of the apple tissues. Several miRNAs were expressed to similar levels in all tissues tested, e.g. miR396 was highly expressed in all tissues and miR162 showed moderate to low levels of expression in all tissues; miR164 and miR393 were barely detectable in the shoot apex, whereas miR160, miR169 and miR390 were more abundant in the shoot apex than other tissues; miR398, miR403 and miR408 appeared up-regulated in leaf. Several miRNAs were differentially expressed across the stem tissues: miR160 showed higher expression in the phloem than xylem and was barely detectable in periderm; miR394 showed higher expression in the xylem than phloem and was barely detectable in periderm; miR168 was more abundant in periderm than phloem and xylem; miR171 was absent from phloem but was detected both in periderm and xylem.

**Table 1:** miRNAs and their predicted targets.

	Conservation status			
		
miRNA family	*Arabidopsis*	*Oryza* (rice)	*Populus* (poplar)	Predicted target gene(s)
miR156	√	√	√	Squamosa promoter-binding proteins[57]
miR159/319	√	√	√	GAMYB transcription factors[57]
miR160	√	√	√	Auxin response factors (ARF) [57]
miR162	√	√	√	DICER-LIKE 1 (DCL1) [57]
miR164	√	√	√	NAC domain transcription factors[57]
miR156/166	√	√	√	HD-ZIP transcription factors[57]
miR167	√	√	√	Auxin response factors (ARF) [57]
miR168	√	√	√	ARGONAUTE 1 (AGO1) [57]
miR169	√	√	√	HAP2-like transcription factors[57]
miR171	√	√	√	Scarecrow-like transcription factors[57]
miR172	√	√	√	APETALA 2 transcription factors[58]
miR390	√	√	√	TAS3[59]
miR393	√	√	√	F-box transcription factors (TIR1) [60]
miR394	√	√	√	F-box transcription factors[60]
miR396	√	√	√	GRF, rhodenase[60]
miR397	√	√	√	laccase[60]
miR398	√	√	√	Copper superoxid dismutase, CytC oxidase[60]
miR403	√	√	√	ARGONAUTE 2 (AGO2)[20]
miR408	√		√	Peptide chain release factor, laccase[20]
miR475			√	PPR proteins[8]
miR476			√	PPR proteins[8]

### Some apple miRNAs are abundant in the phloem tissue

**Figure 2:**
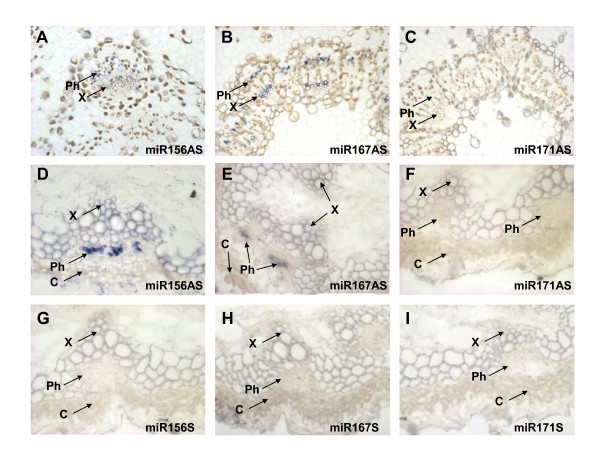
**miRNAs are detected in apple and *Arabidopsis *phloem tissue**. A-C, Spatial expression of miR156, miR167 and miR171 in apple seedling stem; miR156 and miR167, but not miR171, were detected in apple vascular tissue by *in situ *hybridization using appropriate antisense oligonucleotide probes. D-F, Spatial expression of miR156, miR167 and miR171 in *Arabidopsis *inflorescence stem; miR156 and miR167, but not miR171, were detected in *Arabidopsis *phloem by *in situ *hybridization using appropriate antisense oligonucleotide probes. G-I, No hybridisation signal was detected in *Arabidopsis *inflorescence stem using sense oligonucleotide probes. AS, antisense; S, sense; Ph, phloem; X, xylem; C, cortex.

Periderm, phloem and xylem tissues used for RT-PCR were obtained by peeling the layers of the stem and therefore might contain contamination from adjacent cell layers. To distinguish between cell layers, spatial expression of a subset of miRNAs was further analysed using *in situ *hybridisation. Antisense oligonucleotides corresponding to miR156 and miR167 but not miR171 produced a hybridization signal in the phloem of apple seedling stems (Figure 2A-C). Some expression could also be detected around the xylem. To address the universality of phloem-associated expression across species, *Arabidopsis *stems were analysed by *in situ *hybridization. Similar phloem expression for the same subset of miRNAs was detected in the *Arabidopsis *inflorescence stem. Both miR156 and miR167 antisense probes produced a strong hybridization signal in the phloem (Figure 2D-E), while no signal was detected with miR171 antisense oligonucleotide probe (Figure 2F), consistent with the RT-PCR results. In addition, no signal could be detected using corresponding sense probes (Figure 2G-I).

### Apple phloem sap obtained by aphid stylectomy contains small RNA

**Figure 3:**
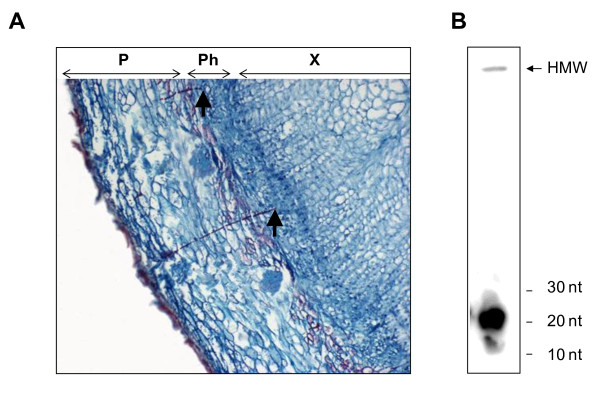
**Woolly apple aphid-derived phloem sap contains small RNA**. A, Woolly apple aphid stylets deposited in apple phloem. The tips of the stylets are indicated by arrowheads. B, Radio-labelled SE RNA contains a prominent small RNA band. Size markers are indicated on the right. The weak band of high molecular weight RNA is indicated by an arrow. P, periderm; Ph, phloem; X, xylem.

Small RNAs, including short interfering RNAs (siRNAs) and miRNAs, were previously detected in the phloem sap of herbaceous species obtained by exudation methods. To establish if small RNAs are present in the phloem sap of woody perennials, woolly apple aphid stylectomy was used to obtain the apple SE content. Woolly apple aphids feed by inserting their stylets into the phloem tissue and sucking up the sap (Figure 3A). RNA isolated from an SE sample collected from a single aphid was radioactively labelled and visualised after separation by PolyAcrylamide Gel Electrophoresis (PAGE). A small quantity of high molecular weight RNA remained close to the well of the gel and a strong band of small RNA (~ 20 nt) was detected in the low molecular weight fraction (Figure 3B).

### miRNAs were detected in apple phloem sap

**Figure 4:**
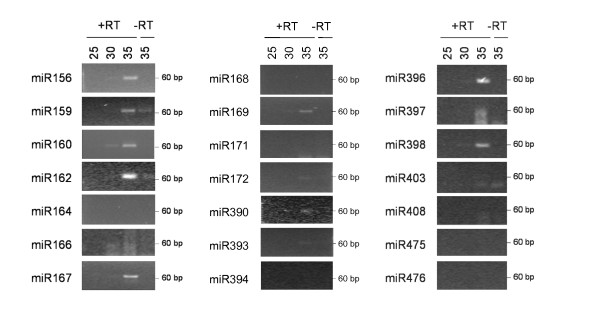
**Expression of miRNAs in apple phloem sap as determined by stem-loop RT-PCR analyses of apple SE contents**. The number of PCR cycles is indicated on the top. +RT, reaction performed with the addition of reverse transcriptase; -RT, minus RT control.

To establish which miRNAs are present in apple phloem translocation stream, RNA purified from the sap derived by aphid stylectomy was used as a template in stem-loop RT-PCR analysis. Because of the very small amounts of sap collected (less than 1 μl per aphid), each RT reaction was performed once per sap collection and repeated two more times for a subset of ten miRNAs using RNA from different sap samples as template. The results between these biological replicates were reproducible. Using this approach miR156, miR159, miR160, miR162, miR167, miR169, miR396 and miR398 were clearly detectable; miR172, miR390 and miR393 produced a weak amplification signal; miR166 and miR397 amplification did not produce the expected product, but resulted in a smear not detected in the minus-RT control; miR164, miR168, miR171, miR394, miR403, miR408 and the miRNAs specific to poplar (miR475 and miR476) were not detected (Figure 4). The summary of miRNAs expressed in apple and detected in apple phloem tissue and apple phloem sap is presented in Table 2.

**Table 2:** miRNAs in the phloem.

	Detected in				Ratio
		
miRNA	apple tissues	apple phloem	apple SE	phloem sap of herbaceous species	Cucurbit phloem sap/VS
miR156	√	√	√	*Brassica*[37]*, c*ucurbits[34]	19.3
miR159	√	√	√	*Brassica*[37]*, c*ucurbits[34]	1.0
miR160	√	√	√	*Brassic*[37]	4.9
miR162	√	√	√	*Brassica*[37]	3.0
miR164	√	√	n.d.	*Brassica*[37]	1.3
miR166	√	√	n.d.*	*Brassica*[37], legumes[35]	34.3
miR167	√	√	√	*Brassica*[37]*, c*ucurbits[34]	18.3
miR168	√	√	n.d.	*Brassica*[37]	16.7
miR169	√	√	√	*Brassica*[37]	24.3
miR171	√	n.d.	n.d.	*Brassica*[37]	0.2
miR172	√	√	√	*Brassica*[37]*, c*ucurbits[34]	3.2
miR390	√	√	√	*Brassica*[37]	56.6
miR393	√	√	√		1.0
miR394	√	√	n.d.		0.3
miR396	√	√	√		0.9
miR397	n.d.	n.d.	n.d.*		7.3
miR398	√	√	√		11.6
miR403	√	√	n.d.		0.4
miR408	√	√	n.d.	*Brassica*[37]	7.4
miR475	n.d.	n.d.	n.d.		n.d.
miR476	n.d.	n.d.	n.d.		n.d.

n.d., not detected; n.d.*, no clear amplification product present

The majority of miRNAs detected in the apple SE sample were previously found in phloem sap samples of herbaceous species, but some miRNAs detected in the phloem sap of herbaceous species were not detected in apple phloem sap (Table 2). This might be the result of contamination during exudation sampling used for herbaceous plants, which is more likely to occur with miRNAs highly abundant in the vascular tissue. To establish the ratio of miRNA accumulation in the phloem sap and vascular tissue, stem-loop RT-PCR expression analysis was performed using RNA purified from pumpkin vascular bundle tissue and phloem sap obtained by the exudation method. A large proportion of miRNAs detected in apple phloem sap were also present in pumpkin phloem sap at significantly higher levels than in the vascular bundles. In contrast, miR171, miR394 and miR403, which were absent from apple phloem sap, were detected in pumpkin phloem sap at a lower level than in the pumpkin vascular bundle. miR168 and miR408 were not detected in the apple SE sample but were enriched in the pumpkin phloem sap sample (Table 2).

### Identification of potential apple miRNA targets

**Figure 5:**
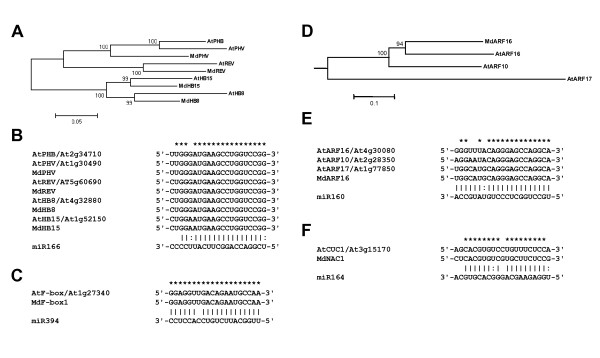
**Apple miRNA targets**. A, Unrooted phylogenetic tree of apple and *Arabidopsis *class III HD Zip transcription factors. B, The miR166 target sites. C, The miR394 complementary sites are identical between *Arabidopsis F-box *and *MdF-box1 *RNA. D, A phylogenetic tree of *Arabidopsis *ARF10, ARF16, ARF17 and MdARF16, rooted against *Arabidopsis *ARF6 protein sequence. E, The miR160 target sites are identical between *Arabidopsis ARF17 *and *MdARF16*. F, Complementary miR164 site in *MdNAC*. *, identical nucleotides in *Arabidopsis *and apple miRNA complementary sequences.

The majority of miRNA molecules chosen for this study and the complementary sites within their mRNA targets display a high degree of conservation between species [62,63]. Thus, the apple expressed sequence tag (EST) database was interrogated for the presence of putative miRNA targets. Gleave et al. [55] previously reported putative orthologues of SPL encoding genes as apple miR156 targets, an apple ARF16 as a miR167 target, orthologues of AP2 and TOE1 as miR172 targets and a copper superoxid dismutase, MdCSD as a miR398 target. In addition to these sequences, putative orthologues of class III HD ZIP transcription factor family were identified from the *Malus *EST database and named MdPHV, MdREV, MdHB8, and MdHB15 (Figure 5A). The miR166 target sites are identical between apple and *Arabidopsis *orthologous genes (Figure 5B) and are highly conserved within plants, with only two nucleotide differences within the whole gene family. Further, a putative orthologue of *Arabidopsis *F-box protein that contains a conserved miR394 target site was identified and named MdF- box1. The *Arabidopsis F-box *and *MdF-box1 *transcripts share the predicted miR394 complementary site that is almost a perfect reverse complement of miR394 (one mismatch only, Figure 5C). A candidate for apple auxin response factor was also identified and named MdARF16. While it seems more closely related to *Arabidopsis *ARF16 and ARF10 proteins (Figure 5D), the miR160 complementary site is identical to that of *Arabidopsis ARF17 *transcript and is a perfect reverse complement of miR160 with a single G/U pair (Figure 5E). An apple NAC-domain protein coding sequence MdNAC1 was identified as a potential miR164 target, the complementary site having three mismatches and two G/U pairs (Figure 5F). The list of apple miRNA target genes analysed in this study with their accession numbers is presented in Additional file 1.

### Expression of miRNAs and corresponding target mRNAs

**Figure 6:**
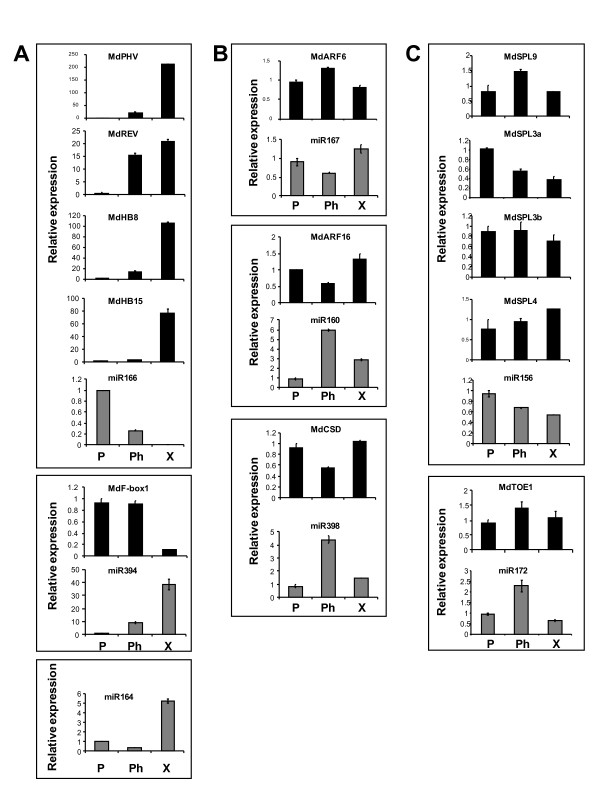
**Expression of miRNAs and their targets in apple stem as determined by stem-loop RT-PCR and standard RT-PCR**. A, Example of miRNAs and their targets that accumulate differentially between stem tissues, with dramatic difference in accumulation levels between tissues, indicative of target exclusion by miRNA action. Predicted miR164 target *MdNAC1 *was not detected in stem tissues. B, Differential expression of miRNAs and minor difference in target accumulation suggest general moderate target reduction. C, No obvious correlation between expression of the miRNA and some of the predicted targets. Real-time PCR reactions were performed in triplicate and the expression was normalised to apple *ACTIN*, with one of the periderm sample replicates acting as calibrator with a nominal value of 1. Error bars represent the means ± SE of three replicate PCRs. The Y axis represents relative expression of individual miRNA or mRNA. P, periderm; Ph, phloem; X, xylem.

To establish the correlation between expression of apple miRNAs and their corresponding targets, RT-PCR and stem-loop RT-PCR were performed using stem tissue RNA. Expression of miRNAs that degrade their target mRNAs shows negative correlation with the respective target mRNAs; they are expressed in adjacent spatial domains and the role of miRNAs is to clear cells of their target gene activities [64] and to maintain sharp gene expression boundaries. Positive correlation is also observed for some miRNAs and a quantitative role for miRNAs was proposed in the reduction of the target mRNA accumulation in cells and tissues that co-express both [65,66]. Accumulation that was over five fold different between tissues was detected for miR166, miR394 and miR164, including total absence of miR166 from xylem. Accumulation of their targets was either not detected in case of miR164 (data not shown) or opposite to that of their corresponding miRNAs, with undetectable or barely detectable accumulation of the target in the tissue with the highest miRNA accumulation (Figure 6A). Differential accumulation of miRNA was detected among tissues for miR167, miR160 and miR398 and their respective predicted targets *MdARF6*, *MdARF16 *and *MdCSD*, but the target fold change was low (Figure 6B). Accumulation of *MdSPL *target transcripts varied among tissues and was not obviously related to differential accumulation of miR156, as was the case for miR172 and *MdTOE1 *(Figure 6C).

## Discussion

### Evolutionary conservation of miRNAs in apple

Mature miRNAs previously detected in *Arabidopsis*, poplar and rice were also detected in apple tissues, with the exception of miR397. The absence of miR397 detection in this study may be due to the limited set of vegetative tissues used, or growth conditions that prevent miR397 accumulation, such as ample copper supply [67]. It is also possible that the apple variant of miR397 has several mismatches to the miR397a sequence chosen for this study and was therefore more difficult to detect using the stem-loop RT-PCR method. Interestingly, the precursor for this sequence could not be identified in the *Malus *EST dataset [55]. A mature miR403, previously detected in *Arabidopsis *and poplar but not in rice, was detected in apple shoot tip, leaf and all stem tissues, confirming conservation between Rosales and Brassicales orders within the Rosid eudicot clade and suggesting that miR403 evolved more recently, after the divergence of monocots and dicots.

Previously described miR475 and miR476 were identified in poplar as miRNAs targeting members of the PPR family and potentially implicated in tree-specific miRNA functions [8]. These sequences were not detected in apple stem tissues, suggesting that these miRNAs evolved quite recently, after the divergence of Malpighiales and Rosales orders within the rosid clade. Indeed, only four similar sequences for miR475 and three similar sequences for miR476 can be found in the miRBase database [68], and they are all identified in poplar. Alternatively, it is possible that the expression of these miRNAs commences in older plants, after substantial secondary growth.

### Phloem tissue and phloem sap are highly enriched for specific miRNAs

Of eighteen miRNA sequences detected in at least one of the analysed apple tissues, all except miR171 were detected in the phloem tissue. Almost half could be detected with fewer than 30 cycles of PCR, suggesting they were relatively abundant in that tissue. These high expression levels are likely to be the result of the abundance of the enzyme responsible for miRNA biogenesis, DCL-1, in the vascular tissue [69].

A number of miRNAs have been previously identified in the phloem sap of several herbaceous plants [34-37] and miR399 was identified as a long-distance signal for the regulation of plant phosphate homeostasis [44,45]. To the best of our knowledge, no data on phloem-mobility of miRNAs were available for woody perennials before this study. Furthermore, all data obtained to date on herbaceous species were derived from exudates collected from cuts, incisions or punctures [70-72]. Phloem sap collected in this manner may contain contamination from outside cells.

The exudate derived from the detached stylets of sap sucking insects such as aphids has been considered the purest form of isolated phloem sap. Stylet exudate is regarded as least subject to artefacts, compared with phloem sap collected by other methods. Therefore, we chose aphid stylectomy as our preferred phloem sap collection method for this study, using woolly apple aphid. Woolly apple aphids feed from apple phloem tissue [73] and their long-term feeding habit at one location and their long stylet bundles facilitate the technology of collecting pure phloem exudates from apple plants (Figure 3A). This study demonstrates that apple phloem sap contains small RNAs that are major contributors to the phloem sap RNA population. Thus, we were able to analyse the sap sample and demonstrate the presence of specific miRNAs in the phloem sap.

To establish further if presence in the phloem sap of a particular miRNA was conserved across species, RNA samples isolated from pumpkin phloem sap and vascular tissue were subjected to stem-loop RT-PCR analysis and the levels of expression were compared. Those miRNAs with significantly higher levels detected in the phloem sap than in the surrounding vasculature are more likely to be non cell-autonomous. Similarly, miRNAs with higher levels detected in the vasculature are more likely to be cell-autonomous. Based on this assumption, more than half the analysed miRNAs showed higher levels of accumulation in the phloem sap. In particular, accumulation of miR156, miR167, miR169, miR390, and miR398 was more than ten-fold higher in the phloem sap than in the vascular tissue, suggesting a possibility of an active mechanism regulating their presence in the phloem sap. All these miRNAs were also detected in the apple SE sample, suggesting some conservation in miRNA non cell-autonomy across plant species. Similarly, miR171, miR394 and miR403, which were not detected in apple phloem sap, were also less abundant in the pumpkin phloem sap sample and, with the exception of miR171, were not cloned from *Brassica napus *phloem sap [37], implying conservation of cell-autonomy for these miRNAs. Some exceptions suggest differences in phloem-mobility of specific miRNAs among species or possibly between herbaceous and woody plants; in particular, miR168 and miR408 accumulated in the phloem sap of pumpkin and *Brassica napus*, but could not be amplified from the apple phloem sap.

### The role of miRNAs in the phloem sap

The presence of small RNAs, including specific miRNAs, in the SE raises the question of their role in the phloem sap. There are two reasons why RNA might be present in the SE. Firstly, some RNA molecules have a role in long-distance signalling. Experiments demonstrating long-distance movements of mRNA through grafted tissue support this role [70,71,74]. It is therefore possible that some miRNAs also acquired a long-distance signalling role. Indeed, a demonstration of shoot-to-root transport and biological activity of shoot-derived miR399 in roots is consistent with the role as a long-distance signal for the regulation of plant phosphate homeostasis [44,45] and the graft-transmissible induction of potato tuberisation by miR172 implies its long-distance signalling role [75]. Secondly, RNA may be dragged by the bulk flow of solutes from companion cell to SEs and therefore reflect the gene expression in the companion cell; for example, the thioredoxin and actin mRNAs were detected in the SEs of rice [52], and a sucrose transporter, an aquaporin, and a proton ATPase mRNA were detected in the SEs of barley [53]. Therefore, miRNAs in the phloem might be accidental outflow from companion cells. However, this hypothesis is not supported by microinjection experiments that exclude diffusion of small RNAs through plasmodesmata and indicate that specific proteins are required to enable transport of a small RNAs through plasmodesmata [34], suggesting a highly regulated mechanism for small RNA transport. In addition, not all miRNAs that were detected in apple phloem tissue were found in the phloem sap, possibly because their movement into the phloem sap is actively prevented. It remains to be determined definitively whether our finding of miRNAs in the apple phloem sap is the result of directed export. At present, the mechanism by which these miRNAs enter the SE and their role in this type of cells cannot be determined. It may be, for instance, that miRNAs in the phloem tissue and sap act to prevent translation and movement of their mRNA targets, the majority of which encode transcription factors, which would have detrimental phenotypic and developmental effects if transported long distance in the phloem. This would be consistent with the finding that phloem sap RNA interferes with translation [76]. Indeed, to date neither of the identified and confirmed miRNA target mRNAs has been shown to move with the phloem across the graft union.

## Conclusions

Conserved families of miRNAs that target genes responsible for plant growth, development, and stress response are differentially expressed in apple tissues. Some of the predicted miRNA target transcripts had low abundance in tissues where miRNAs were detected. Predicted targets of miRNAs detected in the phloem sap usually showed only minor differences in expression levels between tissues. A large proportion of the studied miRNAs are abundant in the phloem tissue and in the phloem sap of both apple and several herbaceous species, with some exceptions suggesting differences in phloem-mobility of specific miRNAs between species.

## Methods

### miRNA sequences

Plant miRNA genes were selected from the Sanger Institute miRBase Sequence Database at http://microrna.sanger.ac.uk/sequences/index.shtml[68].

### Plant material

*Malus *x *domestica *'Royal Gala' (apple) plants were grown at 25 ± 2°C in a greenhouse under natural daylight conditions. A subset of apple seedlings (six months old) was infected with woolly apple aphid (*Eriosoma lanigerum*) and aphid stylectomy was performed once the aphid colonies were established. *Arabidopsis thaliana *'Columbia' plants were grown at 23°C in a greenhouse under natural daylight conditions. *Cucurbita maxima *'Crown' (pumpkin) plants were grown at 23°C in a greenhouse under natural daylight conditions.

### Plant tissue collection

Apple shoot apex, leaf, whole stem, and tissue enriched for epidermis and cortex (periderm), phloem and xylem were collected from six-month-old healthy apple seedlings. Periderm, phloem and xylem tissues were obtained by peeling layers from the apple seedling stem. Pumpkin vascular bundle tissue was obtained by peeling of stem vascular strands.

### Sieve element (SE) sap sampling and handling

Apple SE sap was collected from the stems of intact apple seedlings using micro-cautery techniques to cut the stylets of aphids feeding within the phloem [77]. Seedlings were selected that had aphid colonies feeding from the stem following colonisation over three to four weeks. As aphids colonised fresh tissue other than the stem, they were removed. Just prior to sap sampling, some aphids were also removed from the stem colony until there were approximately 10 feeding aphids on the stem tissue. Removing aphids allowed easier access to the aphid stylets when cauterizing and reduced the sink load on the sieve tubes applied by the feeding aphids. The aphid stylets were cut using a micro-cautery unit http://aphidzapper.com/. Once a stylet had been cut and was freely exuding sap, a droplet of paraffin oil was placed over the stylet. Great care was taken to ensure the exuding sap remained supported solely by the cut stylet and did not come into contact with plant or other aphid material. The sap exudation rate was approximately 3-4 nL s^-1^. The sap collected over the first 20 minutes was discarded, and then sap was collected over the subsequent 1 to 8 h. The SE sample was collected using a glass micropipette constructed using glass capillaries (1 mm O.D; WPI Inc, Sarasota, Florida, USA) and a horizontal tip puller (Cat. No. 2001, Scientific and Research Instruments Ltd, England). The tips of the micropipettes were broken to a diameter of approximately 1-5 μm before being baked at 200°C for 24 hours, cooled, and then back-filled with paraffin oil. An aliquot of RNaseOUT (Invitrogen, Carlsbad, CA) was taken up into the micropipette prior to sap collection and again at the end of sap collection. The collection micropipette was replaced with a fresh paraffin oil- and RNaseOUT-filled micropipette every 45-60 min. All collected sap/RNaseOUT mixture was then pooled into one micropipette and stored at -80°C.

Pumpkin SE sap was collected from well-watered plants using the stem exudation method. Stems were excised with a sterile razor blade and the cut surface was blotted three times with sterile filter paper (3 MM; Whatman, Maidstone, UK). Phloem sap exuded thereafter was collected into microcentrifuge tubes containing 500 μL phenol or TRIzol reagent (Invitrogen), by gently touching the inner wall of the tube. The tube was closed and vortexed well. Several drops were collected in the same tube. The volume of phloem sap collected per tube ranged from 200-400 μL.

### Purification of RNA from solid tissue and RNA gel blot analyses

Apple total RNA was isolated from solid tissue as described by [78]. The concentration of RNA was determined using the NanoDrop ND-1000 Spectrophotometer (NanoDrop Technologies, Wilmington, DE). RNA gel blot analyses of low molecular weight RNA were performed as previously described [34], with the exception that hybridisation was performed at 40°C. The sequences of the antisense and sense probes used for hybridisation are presented in Additional file 2.

### Purification of RNA from phloem sap

Apple phloem sap samples, each containing 0.3-0.6 μL, were diluted to 50 μL using nuclease-free water. An equal amount of phenol-chloroform mix (1:1) was added, the sample was then vortexed, and then centrifuged (16,000 g) for 15 min at 4°C. The aqueous phase was carefully transferred to a fresh tube, an equal volume of chloroform was added and the sample was vortexed and then centrifuged as described above. RNA was then precipitated using 1 volume of cold isopropanol and 0.1 volume of 3 M sodium acetate, with the addition of 20 μg of glycogen (Invitrogen). The samples were subjected to centrifugation (16,000 g) for 60 min at 4°C. The RNA pellet was washed with 70% (v/v) ethanol and resuspended in 2 μL of nuclease-free water.

Pumpkin phloem sap sample collected in 500 μL phenol was vortexed, followed by centrifugation (16,000 g) for 15 min at 4°C. The aqueous phase was carefully transferred to a fresh tube, extracted with phenol-chloroform mix, followed by chloroform and then precipitated as described above. The RNA pellet was re-suspended in 10 μL of nuclease-free water and the RNA was quantified using the NanoDrop ND-1000 Spectrophotometer.

### RNA radio-labelling and visualisation

RNA was treated with DNaseI (Invitrogen) according to the manufacturer's instructions, and subsequently end-labelled using the 5' exchange reaction for 30 min at 37°C [79], using 10 units of T4 polynucleotide kinase (Invitrogen) with the supplied buffer to which was added 100 mM ADP, 2.5 nM ATP, and 165 nM [γ-32P]ATP (10 μCi/μL). Unincorporated 32P-label was removed using ProbeQuant G-50 microcolumns (GE Healthcare, formerly Amersham Biosciences, Buckinghamshire, UK) according to the manufacturer's instructions. For RNA analysis, an equal volume of Loading Buffer II (Ambion, Austin, TX) was added and the sample heated at 94°C for 5 min, followed by electrophoresis on 7 M urea/15% PAGE gel, using 1x TBE (90 mM Tris-borate and 2 mM EDTA) as running buffer. Gels were dried and analysed using a Typhoon scanner (GE Healthcare).

### Reverse transcription-polymerase chain reactions (RT-PCR)

Reverse transcription (RT) was performed using RNA treated with DNase I (Invitrogen), an oligo(dT) primer and the SuperScript III reverse transcriptase (Invitrogen) according to the manufacturer's instructions. Subsequent polymerase chain reactions (PCRs) were performed using the Advantage 2 Polymerase Mix (Clontech) according to the manufacturer's instructions. Products were visualised by agarose gel electrophoresis or quantified using real-time PCR, performed with the FastStart DNA Master SYBR Green I reaction mix (Roche Diagnostics, Manheim, Germany) using the Lightcycler 1.5 instrument and the LightCycler Software version 4 (Roche Diagnostics). Reactions were performed in triplicates and a negative water control was included in each run. Products were quantified against the standard curve using dilutions of a sample with the highest expression and the expression was normalised to apple ACTIN (*MdACT*, accession number CN938023). Error bars shown in the qPCR data represent the means ± SE of three replicate PCR reactions. Primer sequences are presented in Additional file 3. Whenever possible, primers were designed to span the predicted cleavage site in target mRNAs.

Pulsed stem-loop RT and PCR for miRNA detection was performed as described previously [80], using SuperScript III reverse transcriptase (Invitrogen) and Advantage 2 Polymerase Mix (Clontech). Primer sequences were designed according to Chen et al. [81] and are presented in Additional file 4. PCR products were visualised on 4% agarose gels by ethidium bromide staining. Quantifications using real-time PCR were performed following the miRNA SYBR Green I assay protocol [80], on the Lightcycler 1.5 instrument (Roche Diagnostics), using the FastStart DNA Master SYBR Green I reaction mix (Roche Diagnostics), followed by analysis using the LightCycler software, version 4. Reactions were performed in triplicate and a negative water control and minus RT controls were included in each run. The expression was normalised to *MdACT *for apple and PP16 transcript (*CmPP16*) for pumpkin. Error bars shown in the qPCR data represent the means ± SE of three replicate PCR reactions.

### Histology

Apple stem tissue was collected from plants infected with established woolly apple aphid colonies and processed using standard histological methods. In brief, tissue was fixed in formaldehyde-acetic acid-alcohol (FAA) and embedded in wax. Sections 10 μm thick of wax-embedded material were dried onto positively charged slides, de-waxed and stained with safranin fast green [82]. Microscopy was carried out on an Olympus Vanox AHT3 compound microscope with image recording by a Roper Scientific CoolSnap colour digital camera system.

### *In situ *hybridization

Fresh apple seedling stem, shoot tip and *Arabidopsis *inflorescence stem material was fixed in FAA overnight at 4°C. Fixed tissues were dehydrated through an ethanol series and xylene and embedded in paraffin according to Cox et al. [83]. Sections (10 μm) were cut and mounted on Superfrost Plus slides (Biolab Scientific). Sections were rehydrated, prepared for hybridization according to Steel et al. [84], and hybridised overnight. Antisense and sense oligonucleotide probes labelled with digoxygenin (DIG)-11-dUTP were prepared using the DIG Oligonucleotide Tailing Kit (Roche Diagnostics). Hybridisation was performed at 37°C in 30% (v/v) formamide, 4x SSC, 50 μg mL-1 heparin and 100 μg mL-1 polyA solution. After hybridization, the slides were washed as described previously [33], and the probes were visualised as described by Steel et al. [84]. Antisense and sense MdHB15 RNA probes labelled with (DIG)-11-rUTP were transcribed from the full-length cDNA clone with the T7 and SP6 polymerase (Roche Diagnostics). Hybridisation was performed at 48°C in 50% (v/v) formamide, 5x SSC and 50 μg mL-1 heparin. After hybridization, the slides were washed, and the probes were visualised as described [84].

### Phylogeny

Apple EST sequences were uploaded to Vector NTI version 9.0.0 http://www.invitrogen.com. Sequence alignment was performed using Vector NTI Clustal W (opening 15, extension penalty 0.3). Phylogenetic analyses were conducted using MEGA version 3.1 [85] using a minimum evolution phylogeny test and 1000 bootstrap replicates.

## Authors' contributions

EV-G conceived the project, designed the experiments, conducted analyses and prepared the manuscript. NG carried out the apple SE sampling and handling, and contributed to the manuscript. MS and PS conducted analysis of the apple woolly aphid feeding habits. RMM acquired the funding and contributed to and edited the manuscript. All authors read and approved the final manuscript.

## Supplementary Material

Additional file 1 **Apple miRNA targets**. The list of apple miRNA target genes and their accession numbers. Click here for file

Additional file 2 **miRNA hybridisation probes**. The sequences of the antisense and sense probes used for RNA gel blot analyses. Click here for file

Additional file 3 **PCR primers**. The sequences of the oligonucleotides used for real-time PCR quantification. Click here for file

Additional file 4 **miRNA stem-loop RT and PCR primers**. The sequences of the oligonucleotides used for miRNA RT-PCR analysis. Click here for file
