# Supplementary material for: Characterisation of microRNAs from apple (Malus domestica 'Royal Gala') vascular tissue and phloem sap
Source: BMC Plant Biol. 2010 Aug 4;10:159. doi: 10.1186/1471-2229-10-159 (PMC3095296; doi:10.1186/1471-2229-10-159)
Supplement: Additional file 1 — Apple miRNA targets. The list of apple miRNA target genes and their accession numbers. [file 1471-2229-10-159-S1.DOC]

### Additional file 1 – Apple miRNA targets.

| miRNA | Target | Accession number | Best BLASTx *Arabidopsis* hit | Gene name | E value | Reference |
| --- | --- | --- | --- | --- | --- | --- |
|  |  |  |  |  |  |  |
| miR156 | MdSPL3a | EG999281 | AT2G33810 | SPL3 | 2E-37 | Gleave et al., 2007 |
|  | MdSPL3b | EG999262 | AT2G33810 | SPL3 | 2E-33 | Gleave et al., 2007 |
|  | MdSPL4 | EG999265 | AT1G53160 | SPL4 | 1E-42 | Gleave et al., 2007 |
|  | MdSPL9 | EG999266 | AT2G42200 | SPL9 | 1E-67 | Gleave et al., 2007 |
| miR160 | MdARF16 | FJ177422 | AT4G30080 | ARF16 | 0 |  |
| miR164 | MdNAC1 | FJ177423 | AT5G61430 | ANAC/ATNAC5 | 1E-103 |  |
| miR166 | MdPHV | FJ177424 | AT2G34710 | PHB | 0 |  |
|  | MdREV | FJ177425 | AT5G60690 | REV | 0 |  |
|  | MdHB8 | FJ177426 | AT4G32880 | ATHB8 | 0 |  |
|  | MdHB15 | FJ177427 | AT1G52150 | ATHB15 | 0 |  |
| miR167 | MdARF6 | EG999260 | AT1G30330 | ARF6 | 2E-82 | Gleave et al., 2007 |
| miR172 | MdAP2 | EG999282 | AT4G36920 | AP2 | 1E-123 | Gleave et al., 2007 |
|  | MdTOE1 | EG999257 | AT2G28550 | TOE1 | 6E-80 | Gleave et al., 2007 |
| miR394 | MdFbox | FJ177428 | AT1G27340 | F-box family protein | 1E-179 |  |
| miR398 | MdCSD | EG999289 | AT1G08830 | CSD1 | 1E-74 | Gleave et al., 2007 |

### 
