# Supplementary material for: Characterisation of microRNAs from apple (Malus domestica 'Royal Gala') vascular tissue and phloem sap
Source: BMC Plant Biol. 2010 Aug 4;10:159. doi: 10.1186/1471-2229-10-159 (PMC3095296; doi:10.1186/1471-2229-10-159)
Supplement: Additional file 2 — miRNA hybridisation probes. The sequences of the antisense and sense probes used for RNA gel blot analyses. [file 1471-2229-10-159-S2.DOC]

### Additional file 2 – miRNA hybridisation probes.

| miRNA | Description | Sequence |
| --- | --- | --- |
|  |  |  |
| miR156a | miRNA sequence | UGACAGAAGAGAGUGAGCAC |
|  | Antisense probe | GTGCTCACTCTCTTCTGTCA |
|  | Sense probe | TGACAGAAGAGAGTGAGCAC |
| miR167a | miRNA sequence | UGAAGCUGCCAGCAUGAUCUA |
|  | Antisense probe | TAGATCATGCTGGCAGCTTCA |
|  | Sense probe | TGAAGCTGCCAGCATGATCTA |
| miR171a | miRNA sequence | UGAUUGAGCCGCGCCAAUAUC |
|  | Antisense probe | GATATTGGCGCGGCTCAATCA |
|  | Sense probe | TGATTGAGCCGCGCCAATATC |
