# Supplementary material for: Characterisation of microRNAs from apple (Malus domestica 'Royal Gala') vascular tissue and phloem sap
Source: BMC Plant Biol. 2010 Aug 4;10:159. doi: 10.1186/1471-2229-10-159 (PMC3095296; doi:10.1186/1471-2229-10-159)
Supplement: Additional file 3 — PCR primers. The sequences of the oligonucleotides used for real-time PCR quantification. [file 1471-2229-10-159-S3.DOC]

### Additional file 3 – PCR primers.

| Target | Primer | Sequence |
| --- | --- | --- |
|  |  |  |
| *MdPHV* | Forward | GATTTATGAAGCAGCAACTGCATACC |
|  | Reverse | GAATCCGGACCAGGCTTCATCCCA |
| *MdREV* | Forward | CTCTAGAGCTCTCTCTCTTCCTCCT |
|  | Reverse | GAATCCGGACCAGGCTTCATCCCA |
| *MdHB8* | Forward | CACAGCCACTTCTCGACAACTC |
|  | Reverse | GAATCCGGACCAGGCTTCATCCCA |
| *MdHB15* | Forward | GCACATTTGGTTGAGACCCAGTTG |
|  | Reverse | GAATCCGGACCAGGCTTCATCCCA |
| *MdFbox* | Forward | CTGTGGGGATGACTCTGAATAGGA |
|  | Reverse | ATTTGGCATTCTGTCAACCTCCTTCCA |
| *MdNAC1* | Forward | AGGTGTAAAGACCAACTGGGTGA |
|  | Reverse | CGGAGAAGCACGACACGTGAG |
| *MdSPL3a* | Forward | ACTCCTACAGGGAGAAGATCTGG |
|  | Reverse | GCTATGAAACCTGCTACATTGCT |
| *MdSPL3b* | Forward | AGGGCCATGTTGTAAGGTAGATT |
|  | Reverse | GACAACTCCTTTTACGGTCATCA |
| *MdSPL4* | Forward | GAGCTGCAAGTAGACAGGAAGAA |
|  | Reverse | GAGACAAGCACAACCTGAGACTT |
| *MdSPL9* | Forward | CTCTACGAAAACAGCAGCAGAAT |
|  | Reverse | TGTAGGTAAAAGCCGGATGTAGA |
| *MdARF6* | Forward | GGGAGAGAGTGCTCATTAGACCA |
|  | Reverse | ACAAGCTGCCAGCCTGATCTCA |
| *MdARF16* | Forward | ACGCGTTAGCCCATGGTTGGTAG |
|  | Reverse | TGAGCATGCCTGGCTCCCTGCAT |
| *MdCSD* | Forward | ATTCTCCGCTCTCTCTCTCTCTC |
|  | Reverse | GAGGCCAGAGATACATCCAGTC |
| *MdAP2* | Forward | GGACTTCGGCCTAAGCTTAACAT |
|  | Reverse | GGAATCCTGATGATGCTGCAGC |
| *MdTOE1* | Forward | GAGCCTACGGATTTCTCAACCTT |
|  | Reverse | GGAATCCTGATGATGCTGCAATAGA |
| *MdACT* | Forward | TGACCGAATGAGCAAGGAAATTACT |
|  | Reverse | TACTCAGCTTTGGCAATCCACATC |
| *CmPP16* | Forward | GTGGTAAAGGACTTCAAGCCCACGACC |
|  | Reverse | ATGGGTTTGAAGAAGCCAAGCCACTTA |
