# Supplementary material for: Characterisation of microRNAs from apple (Malus domestica 'Royal Gala') vascular tissue and phloem sap
Source: BMC Plant Biol. 2010 Aug 4;10:159. doi: 10.1186/1471-2229-10-159 (PMC3095296; doi:10.1186/1471-2229-10-159)
Supplement: Additional file 4 — miRNA stem-loop RT and PCR primers. The sequences of the oligonucleotides used for miRNA RT-PCR analysis. [file 1471-2229-10-159-S4.DOC]

### Additional file 4 – miRNA stem-loop RT and PCR primers.

| miRNA | Description | Sequence |
| --- | --- | --- |
|  |  |  |
| miR156a | miRNA sequence | UGACAGAAGAGAGUGAGCAC |
|  | RT primer | GTCGTATCCAGTGCAGGGTCCGAGGTATTCGCACTGGATACGACGTGCTC |
|  | Forward primer | GCGGCGGTGACAGAAGAGAGT |
| miR159a | miRNA sequence | UUUGGAUUGAAGGGAGCUCUA |
|  | RT primer | GTCGTATCCAGTGCAGGGTCCGAGGTATTCGCACTGGATACGACTAGAGC |
|  | Forward primer | CGGCGGTTTGGATTGAAGGGA |
| miR160a | miRNA sequence | UGCCUGGCUCCCUGUAUGCCA |
|  | RT primer | GTCGTATCCAGTGCAGGGTCCGAGGTATTCGCACTGGATACGACTGGCAT |
|  | Forward primer | TTCCTTGCCTGGCTCCCTGT |
| miR162a | miRNA sequence | UCGAUAAACCUCUGCAUCCAG |
|  | RT primer | GTCGTATCCAGTGCAGGGTCCGAGGTATTCGCACTGGATACGACCTGGAT |
|  | Forward primer | CGGCGTCGATAAACCTCTGC |
| miR164a | miRNA sequence | UGGAGAAGCAGGGCACGUGCA |
|  | RT primer | GTCGTATCCAGTGCAGGGTCCGAGGTATTCGCACTGGATACGACTGCACG |
|  | Forward primer | TGACGTTGGAGAAGCAGGGCA |
| miR166a | miRNA sequence | UCGGACCAGGCUUCAUUCCCC |
|  | RT primer | GTCGTATCCAGTGCAGGGTCCGAGGTATTCGCACTGGATACGACGGGGAA |
|  | Forward primer | TCGCTTCGGACCAGGCTTCA |
| miR167a | miRNA sequence | UGAAGCUGCCAGCAUGAUCUA |
|  | RT primer | GTCGTATCCAGTGCAGGGTCCGAGGTATTCGCACTGGATACGACTAGATC |
|  | Forward primer | TCGCGTGAAGCTGCCAGCAT |
| miR168a | miRNA sequence | UCGCUUGGUGCAGGUCGGGAA |
|  | RT primer | GTCGTATCCAGTGCAGGGTCCGAGGTATTCGCACTGGATACGACTTCCCG |
|  | Forward primer | GCGGCGGTCGCTTGGTGCAGGT |
| miR169a | miRNA sequence | CAGCCAAGGAUGACUUGCCGA |
|  | RT primer | GTCGTATCCAGTGCAGGGTCCGAGGTATTCGCACTGGATACGACTCGGCA |
|  | Forward primer | GCGGCGGCAGCCAAGGATGACT |
| miR171a | miRNA sequence | UGAUUGAGCCGCGCCAAUAUC |
|  | RT primer | GTCGTATCCAGTGCAGGGTCCGAGGTATTCGCACTGGATACGACGATATT |
|  | Forward primer | TTCCTTGATTGAGCCGCGCC |
| miR172a | miRNA sequence | AGAAUCUUGAUGAUGCUGCAU |
|  | RT primer | GTCGTATCCAGTGCAGGGTCCGAGGTATTCGCACTGGATACGACATGCAG |
|  | Forward primer | CGGCGCAGAATCTTGATGATG |
| miR390a | miRNA sequence | AAGCUCAGGAGGGAUAGCGCC |
|  | RT primer | GTCGTATCCAGTGCAGGGTCCGAGGTATTCGCACTGGATACGACGGCGCT |
|  | Forward primer | CGGCGAAGCTCAGGAGGGAT |
| miR393a | miRNA sequence | UCCAAAGGGAUCGCAUUGAUC |
|  | RT primer | GTCGTATCCAGTGCAGGGTCCGAGGTATTCGCACTGGATACGACGATCAA |
|  | Forward primer | GCGGCGGTCCAAAGGGATCGCA |
| miR394a | miRNA sequence | UUGGCAUUCUGUCCACCUCC |
|  | RT primer | GTCGTATCCAGTGCAGGGTCCGAGGTATTCGCACTGGATACGACGGAGGT |
|  | Forward primer | CGGCGCTTGGCATTCTGTCC |
| miR396a | miRNA sequence | UUCCACAGCUUUCUUGAACUG |
|  | RT primer | GTCGTATCCAGTGCAGGGTCCGAGGTATTCGCACTGGATACGACCAGTTC |
|  | Forward primer | CGGCGTTCCACAGCTTTCTT |
| miR397a | miRNA sequence | UCAUUGAGUGCAGCGUUGAUG |
|  | RT primer | GTCGTATCCAGTGCAGGGTCCGAGGTATTCGCACTGGATACGACCATCAA |
|  | Forward primer | CGGCGTCATTGAGTGCAGCG |
| miR398a | miRNA sequence | UGUGUUCUCAGGUCACCCCUU |
|  | RT primer | GTCGTATCCAGTGCAGGGTCCGAGGTATTCGCACTGGATACGACAAGGGG |
|  | Forward primer | GCGGCGGTGTGTTCTCAGGTCA |
| miR403a | miRNA sequence | UUAGAUUCACGCACAAACUCG |
|  | RT primer | GTCGTATCCAGTGCAGGGTCCGAGGTATTCGCACTGGATACGACCGAGTT |
|  | Forward primer | CGGCGTTAGATTCACGCACA |
| miR408a | miRNA sequence | AUGCACUGCCUCUUCCCUGGC |
|  | RT primer | GTCGTATCCAGTGCAGGGTCCGAGGTATTCGCACTGGATACGACGCCAGG |
|  | Forward primer | CGGCGCATGCACTGCCTCTTC |
| miR475 | miRNA sequence | UUACAGUGCCCAUUGAUUAAG |
|  | RT primer | GTCGTATCCAGTGCAGGGTCCGAGGTATTCGCACTGGATACGACCTTAAT |
|  | Forward primer | CGGCGTTACAGTGCCCATTG |
| miR476 | miRNA sequence | UAGUAAUCCUUCUUUGCAAAG |
|  | RT primer | GTCGTATCCAGTGCAGGGTCCGAGGTATTCGCACTGGATACGACCTTTGC |
|  | Forward primer | CGGCGCTAGTAATCCTTCTTT |
| Universal | Reverse primer | GTGCAGGGTCCGAGGT |
